# Robust Polymer Planar Bragg Grating Sensors Embedded in Commercial-Grade Composites

**DOI:** 10.3390/polym12030715

**Published:** 2020-03-23

**Authors:** Stefan Kefer, Theresia Sauer, Steffen Hessler, Michael Kaloudis, Bernhard Schmauss, Ralf Hellmann

**Affiliations:** 1 Applied Laser and Photonics Group, Aschaffenburg University of Applied Sciences, Wuerzburger Strasse 45, 63743 Aschaffenburg, Germany; 2 Laboratory for Packaging and Interconnection Technology, Aschaffenburg University of Applied Sciences, Wuerzburger Strasse 45, 63743 Aschaffenburg, Germany; 3 Institute of Microwaves and Photonics, University of Erlangen-Nuremberg, Cauerstrasse 9, 91058 Erlangen, Germany

**Keywords:** Bragg grating sensors, carbon fiber reinforced polymers, cyclic olefin copolymers

## Abstract

This contribution demonstrates the functionality of polymer planar Bragg grating (PPBG) sensors integrated into commercial-grade carbon fiber reinforced polymer (CFRP) components. Multiple CFRP specimens are generated by curing a stack of pre-impregnated fibers inside of a heated mechanical press, exposing the polymer sensor to a pressure of 7 bar and a temperature of 120 °C for 2 h. After integration, the sensor still exhibits a strong and evaluable signal. Subsequent flexural experiments reveal a linear response of the integrated sensor’s Bragg wavelength to the CFRP specimen’s maximum deflection. Additional findings demonstrate that the embedded PPBG can be used to detect plastic deformations of a CFRP workpiece, whereas a linear correlation of plastic deformation to the resulting Bragg signal offset is determined. A plausibility check of the obtained results is delivered by a comparison of three-point flexural experiments on bulk CFRP workpieces, without integrated sensors and additional specimens featuring external optical sensors affixed to their surface. It is found that PPBGs based on cyclic olefin copolymers are able to overcome the temperature-related limitations of traditional polymer-based optical sensors and can thus be directly integrated into commercial-grade composites during production.

## 1. Introduction

In recent years, the usage of carbon fiber reinforced polymers (CFRP) for lightweight and high-performance applications, such as the aerospace industry and civil engineering, has increased dramatically, since this material class combines excellent mechanical properties with low weight and density [1,2]. However, their true mechanical behavior cannot be fully represented by classical theories. For example, under bending, the resulting flexural behavior of a CFRP laminate is influenced by various additional effects, such as interlaminar shear stresses, which again, are highly dependent on the employed fiber orientations [3,4,5]. Additionally, in comparison to classical materials, composites tend to behave inherently different in excess load situations. While most fatigue phenomena in metals or polymers can be detected without difficulties, predicting the failure process of CFRPs is a challenging and elaborate task, since even microscopically small fissures can cause sudden and unpredictable failure of a CFRP component [6]. Therefore, immense resources have to be invested for the detection of damage, delamination and material fatigue. While there are several modern and sophisticated approaches for the structural health monitoring of composites [7,8], most damage inspection methods are based on offline testing procedures. Consequently, in order to minimize costs for manufacturers while simultaneously addressing user safety concerns, alternative technologies enabling the in situ detection of structural changes in CFRPs need to be developed.

In previous studies, the integration of silica-based fiber Bragg gratings (FBG) was identified as a promising method for the live evaluation of composites [9,10]. Albeit, their integration into CFRPs has proven to be challenging due to stress-induced birefringence effects [11]. Furthermore, their inherent one-dimensionality needs to be overcome by employing complex sensor structures, such as multicore FBGs, in order to enable absolute quantification of multidimensional deflections [12,13]. Polymer planar Bragg gratings (PPBG), on the other hand, offer up to three-dimensional detection of deformations, simply by evaluating multiple photonic structures on a single substrate [14,15]. Additionally, most polymer substrates offer inherent photosensitivity, whereas silica fibers need to be hydrogen loaded or germanium doped in order to enable constant refractive index modifica-tions [16,17]. Furthermore, polymer-based sensors exhibit increased sensitivity towards mechanical deformations due to their distinctly low Young’s modulus [18]. Missine et al. already demonstrated the integration of epoxy-based planar Bragg gratings into a fiber-reinforced polymer [19]. However, the employed fabrication process is conducted under a pressure of only 500 mbar at a temperature of 85 °C [20], whereas high-grade composite laminates are normally fabricated at pressures of several bar and temperatures beyond 100 °C for an extended period of time [21]. Consequently, the insufficient glass transition temperature of most polymers prevents their successful integration into commercial CFRPs, since they cannot withstand harsh fabrication processes. This challenge can be overcome by employing PPBGs based on cyclic olefin copolymers (COC) which, due to an outstanding glass transition temperature of up to 250 °C, are able to outlast temperature measurements up to 160 °C [22,23]. Additionally, this thermoplastic is inherently unsusceptible towards relative humidity fluctuation, eliminating this undesired cross-sensitivity [24,25]. Thus, they represent a particularly promising sensing technology for CFRP integration. Against this background, this study demonstrates that COC-based PPBGs are able to withstand a pressure of 7 bar and a temperature of 120 °C for a duration of 2 h, during the fabrication process of a high-grade CFRP component. The functionality of the embedded optical sensor is discussed by means of various three-point bending experiments. For the first time, it is demonstrated that the sensor is able to detect and quantify residual plastic deformations induced by excessive loads. Furthermore, a critical analysis of sensor positioning, as well as the embedding process and its impact on the CFRP specimen’s mechanical behavior, is given. Finally, the obtained results are compared with the response of a PPBG affixed to the surface of a reference composite.

## 2. Materials and Methods

**Figure 1:**
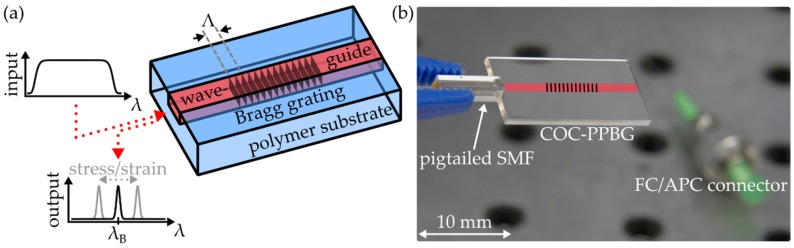
(**a**) Working principle of a polymer planar Bragg grating sensor; (**b**) Polymer planar Bragg grating in a cyclic olefin copolymer substrate (COC-PPBG); positioning of waveguide and Bragg grating are indicated), butt-coupled to a pigtailed standard single-mode fiber.

In principal, PPBGs are constituted of a polymer substrate featuring a waveguide and a Bragg grating, as depicted in Figure 1a.

A Bragg grating is a periodic refractive index modulation which acts as a narrowband reflector for light traversing the waveguide. Its main reflection wavelength, denoted as Bragg wavelength $\lambda_{B}$, is given by its effective refractive index $n_{\mathrm{eff}}$ and its grating period Λ, according to

(1) $$\lambda_{B}{=2n}_{\mathrm{eff}}\Lambda .$$

External forces affect Λ and $n_{\mathrm{eff}}$, resulting in a proportional shift of $\lambda_{B}$, which can be detected and evaluated by means of an appropriate interrogation device.

Simultaneous generation of waveguide and Bragg grating within the COC (TOPAS6017S-04, TOPAS Advanced Polymers, Raunheim, Germany) is realized by irradiating the substrate with ultraviolet radiation generated by a KrF excimer laser (BraggStar Industrial, Coherent Europe B.V., Utrecht, Netherlands). All COC-PPBGs used throughout this study are generated by exposing each substrate to an overall fluence ranging from 100 J·cm^−2^ to 200 J·cm^−2^, whereas the single pulses exhibit a pulse duration of 15 ns and a pulse energy of 68 µJ at a repetition rate of 200 Hz. A detailed discussion of the employed single-writing-step manufacturing method is given by the authors elsewhere [26,27,28]. Figure 1b shows the fabricated COC-PPBG before its integration into a CFRP specimen. In order to evaluate the PPBG, a physical connection to a single-mode fiber (SMF) needs to be established. A UV-curable adhesive (NOA 61, Norland, Cranbury, USA), specified to resist temperatures up to 125 °C, is used to butt couple the optical fiber to the waveguide. Analysis of the reflected sensor signal is done by an industrial-grade interrogation unit (HYPERION si155, Micron Optics, Atlanta, GA, USA), which offers a specified spectral resolution of 1 pm.

Carbon fiber reinforced polymers represent a composite material consisting of at least two components, carbon fibers embedded in a polymer matrix (mostly epoxy resin). While the matrix serves as parent material, the fibers are responsible for redistribution and absorption of external forces. This functional interaction yields components exhibiting outstanding strength-to-weight ratios, whereas the final mechanical properties of CFRPs depend on the used materials. Additionally, the amount and direction of the fibers enclosed within the workpiece play a crucial role. This leads to a distinct anisotropic behavior of single-layer CFRP components, which is usually mitigated by sandwiching layers with alternating fiber orientations [21]. For the manufacturing of commercial-grade CFRPs, it is common to make use of pre-impregnated composite fibers known as prepregs, a compound of semi-cured epoxy with embedded fiber structures. Several prepreg layers are preformed into desired shapes before they are fully cured at high temperatures under immense pressure, thus chemically crosslinking the matrix system to generate operational high-grade CFRP components.

## 3. Sample Preparation and Sensor Integration

Several prepreg layers (P3252S-25, Toray Industries, Tokyo, Japan) are brought to a rectangular shape and subsequently stacked on top of each other to form multiple CFRP specimens. Thereby, every single layer, and thus the resulting specimen, exhibits a fiber orientation of ±45° with respect to its symmetry axis. Afterwards, a heated press is used to cure every workpiece, whereas both plates of the press are actively controlled by a tensile test machine (Autograph AG-X 20kN, Shimadzu, Duisburg, Germany). By this means, a constant pressure of 7 bar is applied to the specimen over a time period of 2 h, at a curing temperature of 120 °C, according and in compliance to an industrial manufacturing process of CFRP workpieces for bicycle components. Altogether, four types of specimens are prepared for subsequent three-point flexural tests. An overview of their composition, dimensions and flexural properties obtained throughout this study is given in Table 1. The flexural modulus $E_{3P}$ specified therein is estimated by employing three-point flexural test data generated throughout the experiments, discussed in this contribution according to

(2) $$E_{3P}=\frac{L^{3}K}{{4\mathrm{bh}}^{3}}$$

where L is the distance in-between both support spans, while b and h are width and thickness of the test sample.

**Figure 2:**
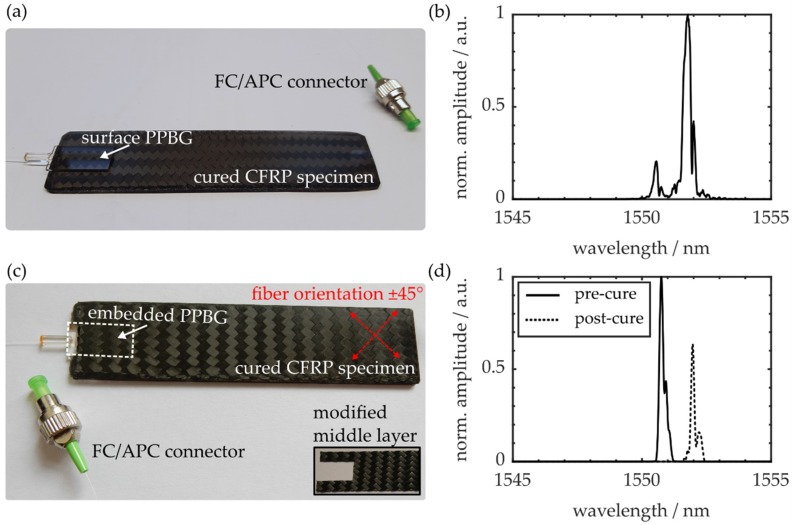
(**a**) Cured CFRP specimen with affixed COC-PPBG and (**b**) sensor signal; (**c**) Cured CFRP specimen with an integrated COC-PPBG; (**d**) Respective sensor signal before and after curing.

The bending stiffness K is represented by the slope of the force-deflection curve, determined by performing multiple three-point flexural tests with different stroke values [29]. Two of the cured CFRP samples and the signal peaks of the respective COC-PPBGs are exemplarily depicted in Figure 2.

Figure 2a shows a specimen with an optical sensor subsequently affixed to its surface, after the workpiece is fully cured and cooled down to room temperature. The corresponding PPBG, fabricated with an overall fluence of 200 J·cm^−2^, exhibits a Bragg wavelength of 1551.63 nm and a spectral full width at half maximum (FWHM) of 298 pm, as demonstrated in Figure 2b. The specimen shown in Figure 2c features an embedded optical polymer sensor, fabricated with an overall fluence of 100 J·cm^−2^. Please note that in order to enable the integration of a PPBG, the central CFRP layers need to be modified by cutting out and removing a section of the respective prepregs, thus generating a cuboidal pocket according to the sensor’s dimensions (20 × 10 × 1 mm³). Figure 2d elucidates that after curing, the integrated sensor exhibits a reduction in signal amplitude of −2 dB and a wavelength shift of −1.236 nm. Whereas the decline in reflection signal can be attributed to changes of the light-guiding properties due to the waveguide-epoxy interaction, the resulting wavelength shift is caused by constant strain induced by the surrounding CFRP material. However, even after being exposed to the harsh and demanding curing process parameters, the polymer sensor evidently delivers a strong and evaluable signal at a Bragg wavelength of 1552 nm with a spectral FWHM of 141 pm.

## 4. Results and Discussion

### 4.1. Specimen A: Flexural Behaviour of Bulk CFRP Specimen

**Figure 3:**
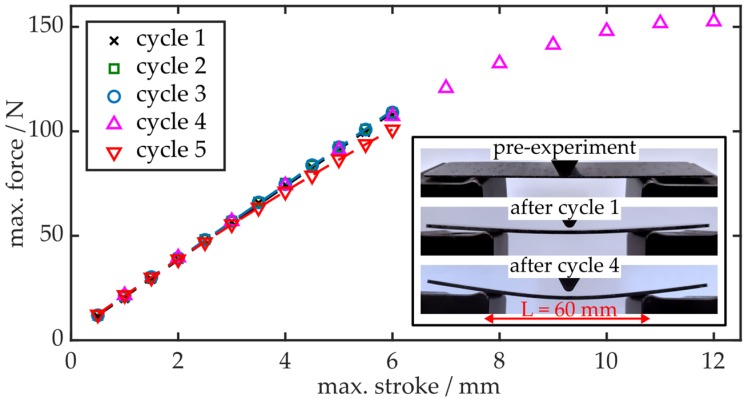
Maximum force as a function of maximum deflection during multiple three-point flexural cycles. The inset shows the sample at the beginning of the experiment and residual deformations after maximum strokes of 6 mm and 12 mm, respectively.

Prior to evaluating the affixed or embedded PPBGs, the flexural properties and behavior of a bulk CFRP specimen are examined by performing multiple three-point flexural testing cycles. Since it does not feature an optical sensor, neither on its surface nor embedded within the CFRP component, the obtained values represent the intrinsic workpiece properties which can thus be used for referencing purposes. Throughout every cycle, the maximum deflection at the center of the sample, also denoted as stroke, is actively controlled by a tensile test machine (EZ-L, Shimadzu, Duisburg, Germany), which simultaneously records the applied loads. In all experiments within this study, the workpiece is bent with a deflection speed of 8 mm·min^−1^ until a predefined value is reached. Subsequently the specimen is kept under maximum stroke for a duration of 20 s before the load is removed at a similar speed, until the sample relaxes completely. As shown in Figure 3, Specimen A is bent to maximum deflections of 6 mm throughout three cycles. Afterwards, it is bent to a maximum stroke of 12 mm in order to purposely compromise its flexural properties by means of excess deformation. A final three-point flexural cycle is used to quantify the resulting reduction of the workpiece’s flexural stiffness.

Throughout cycles 1, 2 and 3, the specimen exhibits a linear force-deflection curve, yielding a stiffness of 17.7 N·mm^−1^ and a flexural modulus of 16.5 GPa. However, as indicated by the inset in Figure 3, even while its flexural properties and thus its structural integrity remains unaltered, a noticeable plastic deformation is observed after the first flexural testing cycle. Increasing the load to achieve strokes larger than 6 mm reveals a non-linear relation between force and maximum deflection. As demonstrated by cycle 5, the excessive deformation within cycle 4 leads to a noticeable stiffness reduction of 9% to 16.1 N·mm^−1^. However, no macroscopic evidence of material failure is detected. The observed behavior of the specimen correlates well to the matrix-dominated failure mechanisms examined in previous studies. Therein, it is shown that failure progression in composite laminates under bending can be described as follows: For small curvatures within the elastic region, deflecting the laminate will yield a linear force-stroke response indicating the CFRP experiences no or negligible structural damage. Increasing the maximum displacement, however, will lead to the formation of matrix micro cracks, which in turn cause a non-linear relation of force and resulting stroke. These micro cracks will then combine to meso cracks, which ultimately induce delamination within the composite, causing a reduction in bending stiffness. Finally, complete failure of the CFRP workpiece occurs due to macro cracks, fiber failure or global loss of stability [30,31,32]. Consequently, within the linear regime up to maximum deflections of 6 mm, the resulting plastic deformation of the specimen cannot be attributed to delamination, but interlayer shear slip effects caused by interlaminar shear forces [33].

### 4.2. Specimen B: Polymer Optical Sensor Affixed to Workpiece Surface

**Figure 4:**
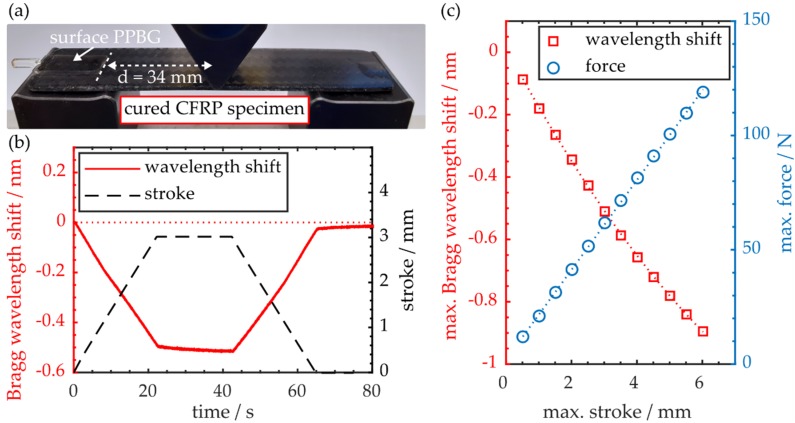
(**a**) CFRP specimen with COC-PPBG affixed to its upper surface in the experimental setup; (**b**) Time trace of workpiece deflection and sensor response during a three-point flexural cycle; (**c**) Maximum Bragg wavelength shift and the respective force as a function of increasing maximum strokes.

Mounting a polymer planar Bragg grating sensor on the outer surface of a CFRP specimen is the most straightforward approach to monitor workpiece deformations during a three-point flexural test. Whereas, from a practical point of view, with this method the sensor lacks protection from environmental mechanical influences, it ensures that the PPBG solely detects resulting deformations of the sample, while the latter is subjected to bending procedures. A physical connection between workpiece and COC-PPBG is established by employing a UV-curable adhesive (NOA 76, Norland, Cranbury, USA). Thereby, the buried waveguide is located underneath the substrate surface which is not in direct contact with the adhesive. Figure 4a depicts Specimen B positioned in the employed three-point flexural setup. Please note that the sensor substrate is located in a distance d of 34 mm to the indenter which induces loads to the central region of the specimen. Consequently, it has sufficient distance to the support span to prevent the unwanted detection of local stress fields.

Figure 4b shows a time trace of a bending cycle with a maximum central workpiece deflection of 3 mm. It demonstrates that bending the workpiece leads to a spectral blue shift of the sensor signal, which can be correlated to local workpiece deformations, although the COC-PPBG is positioned outside the support spans of the experimental setup. The Bragg wavelength drift behavior while the sample is under maximum stroke, as well as the non-linear increase of the maximum Bragg wavelength shift depicted in Figure 4c, indicate a non-ideal mechanical coupling of CFRP and optical sensor by the adhesive film [34]. Evaluation of the resulting force-strain curve obtained by the flexural experiments yields a stiffness of 21.6 N·mm^−1^ and a flexural modulus of 17.1 GPa. Since, in comparison with Specimen A, there is negligible deviation of the flexural modulus, the affixed sensor does not impact the flexural properties of the CFRP workpiece.

**Figure 5:**
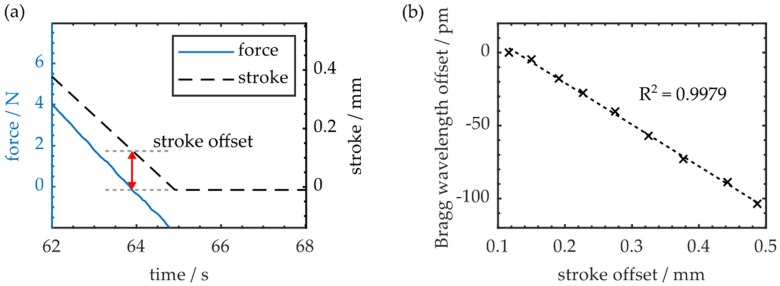
(**a**) Stroke offset due to plastic deformation at the end of a three-point flexural cycle; (**b**) Resulting Bragg wavelength offset as a function of stroke offset.

As already addressed in Section 4.1, the specimen exhibits constant deformation after flexural cycles with maximum strokes larger than 2 mm. As shown in Figure 5a, the stroke offset at the center of the CFRP workpiece is quantified by evaluating the remaining stroke value given by the tensile testing machine, while the external load on the specimen is already zero at the end of a cycle.

It is found that the residual stroke offset increases with the maximum deflection value during a bending cycle. Simultaneously, the remaining Bragg wavelength shift or offset at the end of a cycle (see Figure 4b) increases as well. Correlation of both offsets reveals a linear dependency, as demonstrated in Figure 5b. Therefore, the Bragg wavelength offset can be used as a measure for the plastic deformation of the CFRP specimen, whereas a deformation of 0.49 mm at the center of the workpiece results in a Bragg wavelength offset of −103 pm.

### 4.3. Specimen C: Functionality of Integrated Polymer Optical Sensor

**Figure 6:**
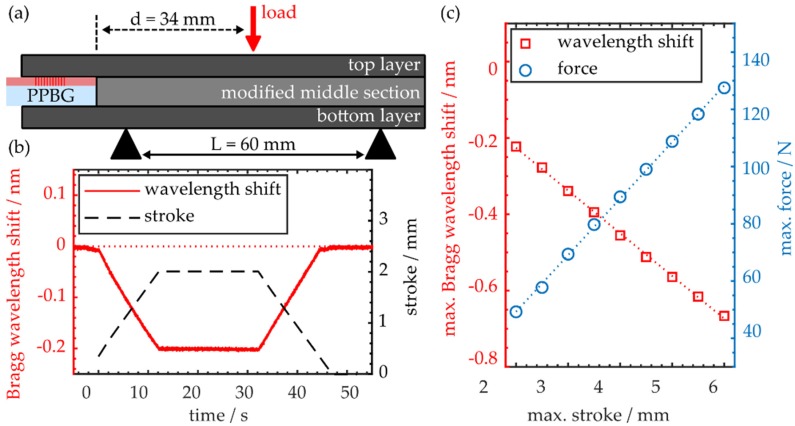
(**a**) Schematic of the employed experimental setup and sensor positioning within the specimen; (**b**) Time trace of workpiece deflection and sensor response during a three-point flexural cycle; (**c**) Maximum Bragg wavelength shift and respective force as a function of maximum stroke during multiple three-point flexural testing cycles.

The functionality of the integrated COC-PPBG is evaluated by using the same three-point flexural test configuration. Figure 6a elucidates the employed setup and the sensor position within the CFRP sample.

In contrast to the surface polymer sensor, the integrated PPBG shows a linear response when correlating the maximum Bragg wavelength shift to the maximum deflection induced to the workpiece, as depicted in Figure 6c. According to linear regression, the sensor response is −112 pm·mm^−1^. However, the overall wavelength shift is reduced, which can partly be explained by the position of the sensor since the actual sensitive element, the Bragg grating structure, is located closer to the center of the workpiece. On the other hand, it is worthwhile to note that modifying the middle section of the specimen in order to form a sensor pocket possibly has a negative impact on the sensor response. Despite this, simultaneous evaluation of the force necessary to deflect the sample yields a bending stiffness of 19.8 N·mm^−1^, which results in a flexural modulus of 16.7 GPa. Since this value is comparable to the flexural moduli of specimens A and B, it can be stated that integrating the sensor has negligible impact on the specimen’s flexural properties in the outline of the conducted experiment. Since the sensor is embedded within the workpiece, there is no drift behavior while the workpiece is held at maximum deflection, as demonstrated in Figure 6b. Therein, it is also demonstrated that up to maximum workpiece deflections of 2 mm, the Bragg wavelength returns to its initial value at the end of a cycle.

**Figure 7:**
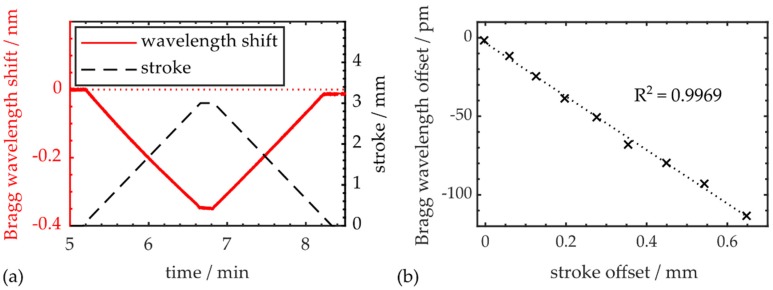
(**a**) Time trace of workpiece deflection and sensor response during three-point bending, revealing a wavelength offset at the end of the cycle; (**b**) Bragg signal offset due to plastic deformation induced by maximum strokes up to 6 mm.

However, as observed during the evaluation of the surface PPBG in Section 4.2, for maximum strokes larger than 2 mm, the Bragg wavelength no longer returns to its initial value at the end of a cycle. A time trace of a bending cycle with a maximum stroke of 3 mm is shown in Figure 7a.

Again, Bragg wavelength and stroke offset increase linearly with the maximum load applied to the workpiece during a flexural testing cycle. Correlating both offsets with each other, as shown in Figure 7b, underlines their relation, since linear regression via MATLAB results in an R² value of 0.9969. Conclusively, it is feasible to use the integrated COC-PPBG to evaluate the CFRP specimen’s plastic deformation or wear. Subsequent to a maximum deflection of 6 mm, the residual stroke offset is 0.64 mm, while the residual Bragg wavelength offset is −113 pm, respectively.

### 4.4. Specimen D: Influence of Workpiece Orientation and Sensor Positioning

**Figure 8:**
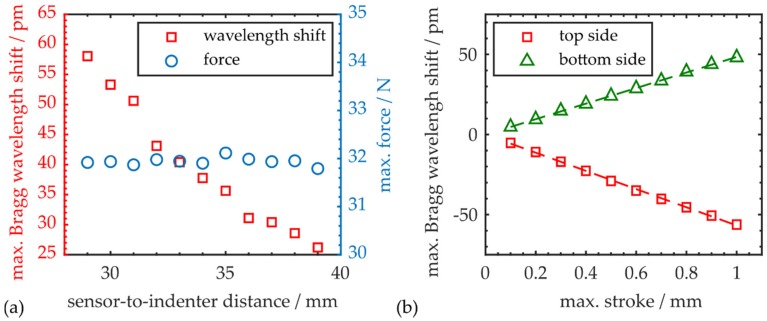
(**a**) Absolute maximum Bragg wavelength shift and force for maximum deflections of 0.5 mm at various sensor-to-indenter distances; (**b**) Sensor signal as a function of maximum stroke for both workpiece orientations, which means the Bragg grating is positioned near the specimen’s top or bottom side, respectively.

A fourth type of specimen, equipped with an integrated optical sensor, is fabricated according to Section 3. In order to vary its mechanical properties, this workpiece features an additional unmodified top and bottom prepreg layer (see Table 1). However, the employed three-point flexural testing setup remains unaltered to guarantee comparable test conditions. In order to assess the integrated sensor’s performance as a function of its distance to the point of maximum workpiece deflection, where external load is induced to the sample by the indenter of the tensile testing machine, the CFRP specimen is laterally repositioned within the setup multiple times. At every position, the sample is bent to a maximum stroke of 0.5 mm. Figure 8a depicts the maximum of the absolute Bragg wavelength shift and the applied force at several sensor-to-indenter distances d, within a range of 10 mm (see Figure 6a).

A minimum distance d of 29 mm is chosen to ensure that the PPBG reacts solely to deformations caused by the bending process. While the force necessary to bend the sample remains constant over the examined range, the resulting sensor response changes drastically, with varying test sample positioning. Since the embedded polymer sensor evaluates only local deformations, it delivers an increased response, while it is positioned closer to the region of maximum deflection. With increasing distance, the impact on the Bragg grating is reduced which translates to a reduced wavelength shift. Conclusively, the positioning of an optical sensor plays a crucial role if its signal is supposed to be correlated with the maximum deflection of the workpiece.

Since as of yet, the vertical position of the Bragg grating structure is located above the central region of the specimen in all experiments, the sensor performance in reverse orientation shall be examined by flipping over the sample. This entails that the sensitive grating structure is now located underneath the middle prepreg layer close to the bottom side. As indicated by Figure 8b, bending the CFRP workpiece leads to a spectral blue shift of the Bragg wavelength, with a response of −56 pm·mm^−1^ if the Bragg grating is located at the top side of the sample. However, if the grating is located near the bottom side, a spectral red shift with a gradient of 48 pm·mm^−1^ is observed. This coincides with the fact that, while top layers are laterally compressed, bottom layers of a bent CFRP specimen are elongated. Thus, depending on its position within the sample, the grating period of the PPBG changes accordingly under the influence of external loads. Minor orientation-dependent deviations in sensor response can be explained by positioning uncertainties, since residual local deformations detected by the sensor also strongly depend on its absolute vertical position within the workpiece. However, the findings demonstrate that a single integrated COC-PPBG enables absolute quantification of deflections in both directions. Additionally, Figure 8b underlines that the observation of central strokes as small as 0.1 mm is possible, even with a sensor-to-indenter distance of several centimeters and with the PPBG being positioned outside the support spans of the three-point flexural setup.

## 5. Conclusions

In conclusion, this study demonstrates that COC-based PPBGs can successfully be integrated into commercial-grade CFRP components. Despite a reduction in signal amplitude after curing of the specimen at a pressure of 7 bar and a temperature of 120 °C for 2 h, the sensor still exhibits a strong and evaluable reflection signal with a Bragg wavelength of 1552 nm and spectral FWHM of 0.14 nm. According to the authors’ best knowledge, this is the first assessment of a polymer-based optical sensor integrated into a composite structure under environmental influences this harsh. The general functionality of the embedded COC-PPBGs is proven by conducting multiple three-point flexural experiments. First, the flexural properties of a bulk CFRP specimen without an optical sensor is examined in order to exclude possible influences of delamination effects. Subsequently, several flexural tests are conducted on a CFRP sample with a PPBG affixed to its surface and a specimen featuring an integrated optical sensor. In both cases, even with the sensor being positioned outside the support spans of the three-point flexural setup, a direct correlation of workpiece deflection and the resulting Bragg wavelength shift is found, whereas the embedded sensor exhibits a linear response of −112 pm·mm^−1^. Additionally, the sensor is able to detect residual plastic deformations attributed to interlayer shear slip effects, whereas a Bragg wavelength offset of −113 pm is caused by a central deformation of 0.64 mm after exposing the workpiece to a maximum stroke of 6 mm. Linear regression of increasing stroke and residual Bragg wavelength offsets yields an R^2^ value of 0.9969, underlining their unambiguous relationship. The observed wavelength offset after each bending cycle can thus be used as a measure for the composite’s wear due to excessive loads. Therefore, employing cyclic olefin copolymers as substrate material for PPBGs provides access to an entirely new application area for polymer-based optical sensors.

## Figures and Tables

**Table 1:** Data of cured carbon fiber reinforced polymer (CFRP) specimens.

Specimen	A	B	C	D
**overall layers**	9			11
**modified middle layers**	/	/	7	7
**COC-PPBG**	/	surface	integrated	integrated
**length/mm**	106.8	106.2	100.0	100.0
**width/mm**	23.5	24.4	22.8	24.1
**thickness/mm**	1.35	1.41	1.40	1.71
**stiffness/N·mm^−1^**	17.7	21.6	19.4	27.1
**flexural modulus/GPa**	16.5	17.1	16.7	12.1
